# 

**Published:** 1909-04-15

**Authors:** 


					﻿Dental
Register
NELVILLE S. HOFF, Editor,
Ann Arbor, Mich.
Vol. LXIII —APRIL, 1909- No. 4
SAMUEL A. CROCKER & CO., Publishers
OHIO DENTAL AND SURGICAL DEPOT
35 W. FIFTH STREET, CINCINNATI, 0.
PRICE, ONE DOLLAR A YEAR
Entered at the Postoffice at Cincinnati, O, as second-class mail matter
				

